# Epigenetic aging in the tumor microenvironment: mechanisms and implications for immunotherapy

**DOI:** 10.3389/fragi.2026.2008892

**Published:** 2026-10-27

**Authors:** Fusako Takezawa, Yohei Sato

**Affiliations:** 1 Immunology and Allergy Research Unit, Department of Otorhinolaryngology, Head & Neck Surgery, Faculty of Medical Sciences, University of Fukui, Fukui, Japan; 2 Division of Molecular Genetics, Research Center for Medical Sciences, The Jikei University School of Medicine, Tokyo, Japan

**Keywords:** cancer immunotherapy, cancer-associated fibroblasts, cellular senescence, epigenetic aging, immunosenescence, tumor microenvironment

## Abstract

Aging reshapes the tumor microenvironment (TME) by altering the immune, stromal, and vascular compartments, yet these changes are not fully explained by chronological age. Epigenetic mechanisms, including DNA methylation, histone modifications, chromatin accessibility, and non-coding RNA programs, may contribute to persistent alterations in cellular function and communication. In this review, we synthesize current evidence on epigenetic remodeling in the aged TME, distinguishing established findings in aging and cancer from mechanisms that remain to be demonstrated specifically in aged tumors. We highlight how epigenetic changes intersect with cellular senescence, chronic inflammation, and metabolic stress to weaken antitumor immunity and influence treatment response, with particular attention to T-cell dysfunction, regulatory T-cells, myeloid cells, fibroblasts, and endothelial cells. We also discuss the limitations of bulk-tissue epigenetic clocks and the emerging value of cell-resolved and spatial analyses. Finally, we consider therapeutic implications, including epigenetic therapies, senescence-directed interventions, and selective targeting of suppressive immune-stromal interactions, while noting that their ability to reverse TME aging remains uncertain. Integrating longitudinal human studies with functional experiments will be essential to determine whether epigenetically altered immune-stromal niches can serve as biomarkers or therapeutic targets beyond chronological age alone.

## 1 Introduction: aging is a property of the TME, not only of the host

Aging is accompanied by progressive changes in cellular and tissue functions (Lopez-Otin et al., 2023). Chronological age provides a straightforward measure of elapsed time, whereas biological and epigenetic ages capture differences in aging across individuals and tissues (Hamczyk et al., 2020). Biological age is estimated from functional and molecular characteristics rather than from chronological age alone. The most common way to measure epigenetic age is with DNA methylation-based models, known as epigenetic clocks (Horvath, 2013). Histone modifications and chromatin remodeling also contribute to age-associated epigenetic changes; however, they are not interchangeable with conventional DNA methylation clocks (de Lima Camillo et al., 2025; Lu et al., 2026). Neither biological nor epigenetic age has a universally accepted measure applicable to all tissues and disease contexts (Lu et al., 2023). Their context dependence and heterogeneity nevertheless offer opportunities to understand how genetic and environmental factors shape human aging.

In addition to age-associated changes, tumors contain cells exposed to oncogenic, inflammatory, and treatment-related stresses (Campisi et al., 2011). Oncogene-induced senescence can act as a cell-intrinsic barrier to malignant transformation by imposing stable growth arrest in premalignant cells (Liu et al., 2018). Chemotherapy and radiotherapy can also induce senescence in tumor cells and surrounding normal tissues (Luo et al., 2025). However, cellular senescence and organismal aging are distinct, although overlapping processes: senescence can occur in young individuals, and not all aged cells are senescent. The proliferative history, metabolic demands, and local environmental stressors differ among cell types; therefore, the heterogeneous components of the tumor microenvironment (TME) do not necessarily age in the same way (Fane and Weeraratna, 2020).

This review examines how age-associated DNA methylation, chromatin, and non-coding RNA programs reshape immune and stromal cell states in tumors, and how these changes influence immune surveillance and treatment response beyond chronological age alone (Figure 1). A central consideration is whether current evidence demonstrates an aging-dependent mechanism within tumors or instead reflects related processes in normal aging, cellular senescence, or cancer independently.

**FIGURE 1:**
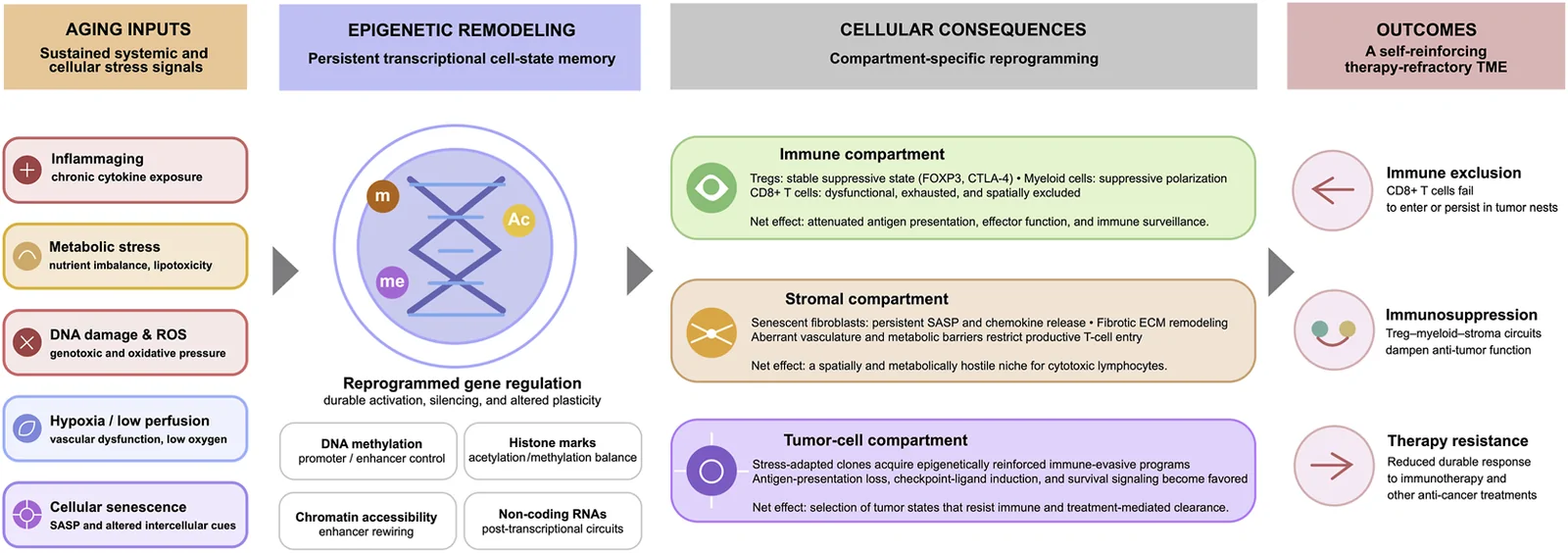
Aging signals reshape the tumor microenvironment through epigenetic cell-state remodeling. Conceptual framework showing how persistent age-associated inputs reprogram immune, stromal, and tumor compartments to favor immune escape and therapeutic failure.

## 2 How to define and measure epigenetic aging in tumors

Cancer cells frequently bypass normal limits of replication, and their epigenetic trajectories may differ substantially from those of non-malignant cells. DNA methylation-based clocks can be applied to tumor samples; however, cancer-associated methylation abnormalities complicate interpretation (Yu et al., 2020). A tumor may appear epigenetically older or younger depending on the clock, tissue, and underlying biology. Consequently, clocks trained on normal tissue cannot be automatically interpreted as measures of functional aging in malignant cells.

Bulk tissue measurements further combine the methylation profiles of cancer cells, infiltrating immune cells, fibroblasts, endothelial cells, and other components (Bell et al., 2019). Differences in tumor purity or cell composition can therefore alter the estimated epigenetic age without indicating accelerated aging in a specific population. Tumor purity, cellular composition, and the tissue used to train the clock should be considered when interpreting these measurements. Chronological and epigenetic ages describe different properties, and their utility should be evaluated against defined outcomes rather than ranked by a single concept of accuracy.

Histone modifications influence DNA packaging and gene regulation, and their dysregulation can activate cancer-associated programs or silence tumor suppressors (Audia and Campbell, 2016). Chromatin accessibility determines the availability of regulatory DNA for transcription factors and other nuclear proteins. Cancer-associated changes in accessible promoters and enhancers provide an additional view of altered regulatory states (Corces et al., 2018). Histone modifications and chromatin accessibility are closely connected; for example, histone acetylation can favor accessible chromatin, although its effects depend on the genomic and cellular context. Non-coding RNAs add another regulatory layer by influencing gene expression and interactions with chromatin-modifying machinery (Anastasiadou et al., 2018).

Advances in single-cell and low-input profiling have enabled the examination of these features in defined cell populations (Frangieh et al., 2026). However, accessibility or transcriptional signatures associated with aging have not yet been validated as epigenetic clocks. Similarly, senescence should be assessed using multiple complementary features, such as persistent growth arrest, DNA damage responses, and secretory changes, rather than a single marker. Combining molecular measurements with functional assays is therefore essential to distinguish an aging-associated signature from a mechanism that drives dysfunction. Due to this limitation, it was not possible to fully distinguish age-associated epigenetic changes from tumor-induced alterations.

## 3 Epigenetic aging of antitumor immune cells

Immune cells exhibit age-associated changes that differ by cell type (Table 1), with T-cell aging being among the most extensively studied examples (Mittelbrunn et al., 2021; Chen et al., 2024). Aging of the adaptive immune system is associated with impaired responses to new antigens and altered immune regulation, contributing to increased susceptibility to infection, reduced vaccine responses, and chronic inflammation. Experimental evidence also indicates that T-cell-intrinsic mitochondrial dysfunction can promote systemic inflammation and premature aging phenotypes in mice (Desdin-Mico et al., 2020). These findings support a role for immune dysfunction in tissue aging, although they do not establish the same causal sequence in human tumors.

**TABLE 1:** Evidence of aging-associated epigenetic remodeling across TME compartments.

TME compartment	Aging-associated epigenetic change	Functional consequence	Evidence in aged tumors	Implications for immunotherapy	Key knowledge gap
CD8^+^ T cells (Chen et al., 2024)	Distinct chromatin-accessibility program in tumor-infiltrating age-associated dysfunctional cells, separable from canonical exhaustion	Impaired effector differentiation and antitumor activity in aged hosts	Direct epigenomic and functional evidence in aged tumor-bearing mice; human epigenetic validation remains limited	Restoring priming and supportive myeloid signals may improve immunity; implications for checkpoint responsiveness require further testing	Which chromatin changes cause dysfunction, are reversible, and predict treatment response?
Tregs (Morales-Nebreda et al., 2021)	Age-associated DNA methylation changes in nonmalignant lung Tregs; EZH2/H3K27me3-dependent regulation in tumor Tregs without an aging comparison	Altered repair programs with aging; maintenance of tumor-associated suppression in separate cancer studies	The cited studies do not establish an age-dependent epigenetic mechanism in intratumoral Tregs	Treg-directed epigenetic modulation has preclinical support; its age-specific therapeutic value remains hypothetical	Does aging alter tumor Treg stability, suppression, or cytokine responsiveness through defined epigenetic mechanisms?
NK cells (Roy et al., 2023)	Age-associated DNA methylation differences in purified circulating human NK cells	Functional effects of individual methylation changes remain unresolved; impaired NK–DC interactions occur in aged tumor models	NK-cell transfer improves anti-PD-L1 responses in aged mice, without demonstrating epigenetic rejuvenation	NK-cell supplementation may restore DC recruitment and T-cell priming; an epigenetic mechanism of benefit remains unproven	Which changes reflect aging *versus* CMV exposure, subset redistribution, or tumor-induced reprogramming?
Myeloid cells, including DCs and macrophages (Roy et al., 2023)	Age-associated DNA methylation in circulating monocytes and granulocytes; applicability to individual tumor-associated subsets remains uncertain	Impaired cDC1-mediated priming in aged tumors; a causal connection to myeloid epigenetic changes remains unresolved	Functional defects and therapeutic rescue are demonstrated in aged mice; the cited studies do not establish the underlying myeloid epigenetic mechanism	Restoring cDC1 activity can improve CD8^+^ T-cell immunity in aged mice, without establishing reversal of epigenetic aging	Which alterations are cell-intrinsic, subset-specific, or imposed by the aged environment?
CAFs (Kaur et al., 2019; Angarola et al., 2025)	Age-related chromatin-accessibility changes in fibroblasts from healthy mammary tissue; relevance to established CAF states remains uncertain	Altered fibroblast identity and matrix programs; separate melanoma studies demonstrate age-dependent ECM remodeling and altered immune-cell motility	Functional stromal-aging evidence exists; the cited studies do not directly connect aged CAF epigenomes to immune exclusion	Stromal or matrix interventions may improve immune access; targeting CAF epigenetic aging remains a proposed strategy	Are age-associated CAF states epigenetically maintained, distinct from senescence, and causally linked to treatment resistance?
Endothelial cells (Honda et al., 2021; Ecker et al., 2019)	Age-associated chromatin changes in nonmalignant vascular cells; increased VCAM1-associated H3K4me3 in experimental endothelial senescence	Increased inflammatory responsiveness in senescence models; age-related lymphatic permeability changes provide separate evidence in melanoma	The cited studies do not establish endothelial epigenetic aging as the cause of vascular dysfunction in aged tumors	Vascular interventions could affect immune trafficking; epigenetic rejuvenation as their mechanism remains untested	How do physiological aging, induced senescence, and tumor-associated stress differ in their effects on endothelial chromatin and trafficking?

Abbreviations: CAF, cancer-associated fibroblast; cDC1, conventional type 1 dendritic cell; CMV, cytomegalovirus; DC, dendritic cell; ECM, extracellular matrix; NK, natural killer; TME, tumor microenvironment.

CD8^+^ T cells progressively lose proliferative reserve and undergo age-related changes in differentiation and memory programs. Aging CD8^+^ T cells transition from self-renewing progenitor states into dysfunctional, terminally exhausted states during chronic antigen exposure or advanced age (Miller et al., 2019). Exhausted CD8^+^ T cells span a broad spectrum of differentiation states. At one end are progenitor exhausted T cells (Tpex), which express TCF1 (encoded by TCF7) and SLAMF6. These cells retain their ability to self-renew, proliferate, and respond to immune checkpoint therapy. Through intermediate stages, they progressively lose these progenitor traits and transition into terminally exhausted T cells. This terminal state is defined by TCF1 loss, persistent expression of inhibitory receptors (PD-1, TIM-3, LAG-3), and rewritten TOX- and EOMES-associated transcriptional profiles. Although terminally exhausted cells rarely proliferate, they can maintain cytotoxic functions. In older individuals, chronic antigen exposure often causes these exhausted cells to accumulate, potentially weakening immune defenses. Crucially, T cell exhaustion differs from cellular senescence; its impact on age-related declines in viral clearance and vaccine responses depends heavily on the specific context.

Human immune-aging studies have identified altered chromatin accessibility in CD8^+^ T cells, providing a direct link between aging and epigenetic remodeling (Ucar et al., 2017). Within tumors, persistent antigen exposure additionally drives exhaustion. Aging, senescence, and exhaustion should not be used synonymously, as they have distinct initiating conditions and only partially overlapping features (Yu et al., 2026). In chronic viral infection models, exhausted T cells retain a distinct epigenetic state despite transient functional reinvigoration by PD-1 blockade (Pauken et al., 2016). This finding provides a rationale for investigating whether age-associated remodeling further restricts T-cell plasticity in tumors, although direct testing is required.

Regulatory T cells (Tregs) also undergo age-associated changes in abundance and function (Jagger et al., 2014). In mice, aging impairs the capacity of Tregs to support central nervous system remyelination, and aspects of this defect can be reversed in a young environment (de la Fuente et al., 2024). These observations illustrate the context dependence of Treg aging. In human, reduced tissue repairing capacity is observed in the aged human Treg after respiratory infection (Morales-Nebreda et al., 2021). However, they should not be interpreted as evidence that aged tumor-infiltrating Tregs uniformly lose their suppressive activity. In the TME, Treg abundance, suppressive function, and tissue repair capacity should be evaluated separately.

Natural killer (NK) cells, B cells, and dendritic cells also show age-associated alterations (Agrawal and Gupta, 2011; de Mol et al., 2021; Qi and Liu, 2023; Roy et al., 2023). Changes in cytotoxicity, B-cell subset composition, antibody responses, antigen uptake, migration, and antigen presentation may weaken coordinated antitumor immunity. NK cells are also influenced by the tumor microenvironment and undergo functional reprogramming into regulatory or dysfunctional phenotypes (Ma et al., 2025). Aging also alters myeloid-cell differentiation and inflammatory responses, contributing to chronic low-grade inflammation (Bleve et al., 2023). However, inflammatory activity does not necessarily imply effective tumor control. Determining which epigenetic changes sustain suppressive rather than protective immune states remains a priority, particularly because direct evidence in aged human tumors is less developed than evidence from peripheral blood or experimental models.

## 4 Aging of stromal and vascular compartments

Age-associated changes in stromal cells represent a critical dimension of TME remodeling (Figure 2). Fibroblasts maintain extracellular-matrix organization and tissue integrity, yet aging can alter their proliferative, reparative, and secretory functions. Studies of dermal fibroblast senescence provide useful examples of these changes (Nan et al., 2025), although findings in the skin cannot be generalized to all tumor-associated fibroblasts. Cancer-associated fibroblasts (CAFs) are heterogeneous, and an activated CAF phenotype does not necessarily indicate senescence or advanced epigenetic age.

**FIGURE 2:**
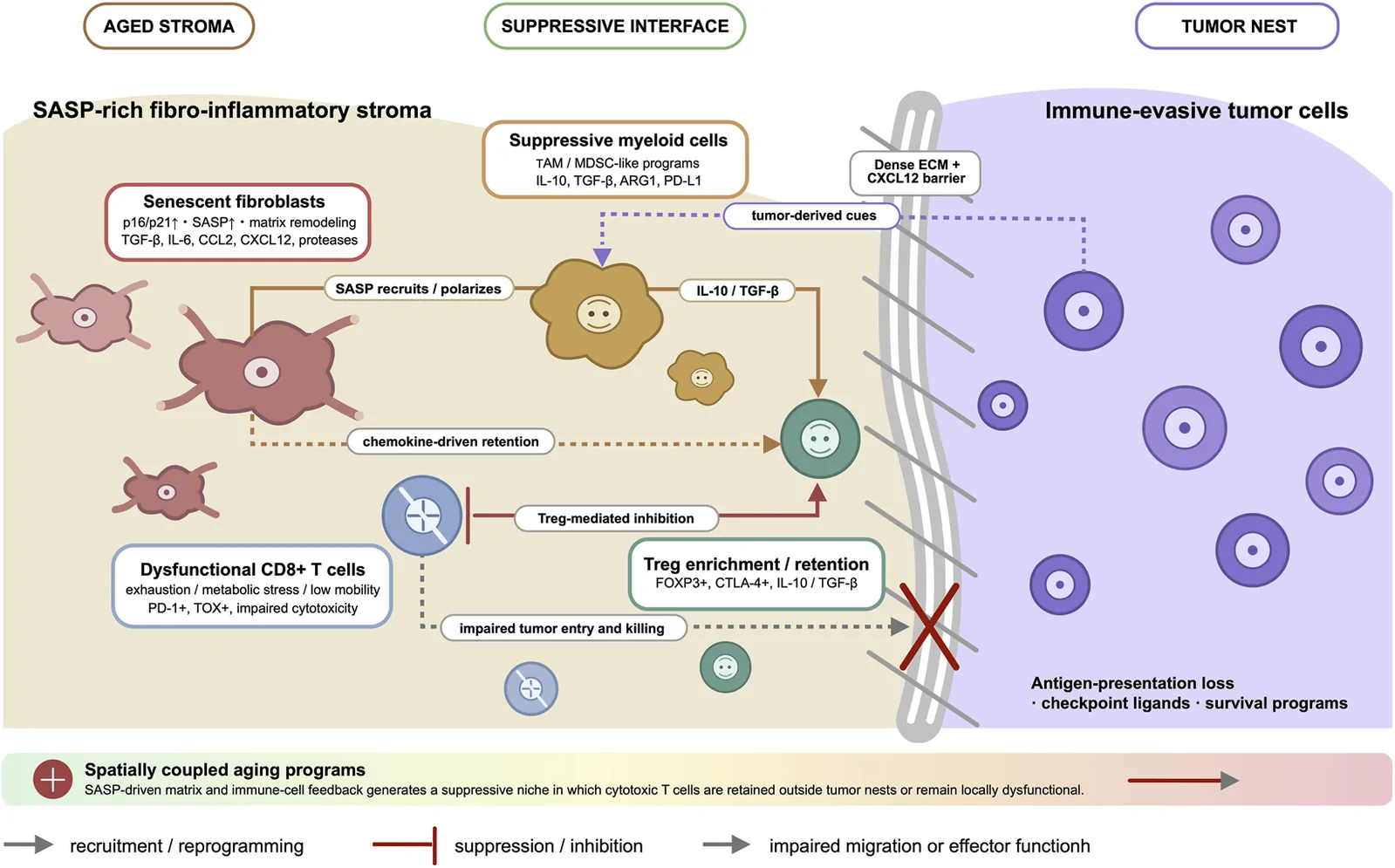
Spatial aged-TME niche: senescent fibroblasts establish a suppressive niche. Conceptual spatial model of intercellular circuits and physical barriers that can promote local immune suppression and treatment resistance in aged tumors.

Senescent fibroblasts release cytokines, chemokines, growth factors, and matrix-remodeling enzymes, collectively termed the senescence-associated secretory phenotype (SASP) (Ghosh and Capell, 2016). In experimental models, stromal senescence has been shown to promote an immunosuppressive microenvironment by recruiting suppressive myeloid cells (Ruhland et al., 2016). Altered matrix organization may also affect immune cell movement and access to tumor cells (Kaur et al., 2019). On the other hand, senescent cells also produce damage-associated molecular patterns (DAMPs) which could promote anti-tumor effects (Yan et al., 2025). These findings support the investigation of senescent stromal niches; however, neither matrix accumulation nor SASP-like transcriptional signatures alone establish epigenetic aging.

Vascular aging involves changes in vascular structure, endothelial function, and regenerative capacity (Moriya and Minamino, 2017; Brandes et al., 2005; Honda et al., 2021). Experimental studies indicate that vascular aging and atherosclerosis can modify tumor growth, angiogenesis, and treatment responsiveness, with effects that are not uniformly tumor-promoting (Klement et al., 2007). Within tumors, endothelial dysfunction and abnormal perfusion may influence hypoxia and immune cell trafficking (Ecker et al., 2019). Age-related endothelial changes should therefore be distinguished from vascular abnormalities induced by the tumor itself.

The central epigenetic question is how persistent regulatory changes sustain stromal and vascular phenotypes. Histone modifications and chromatin remodeling may maintain inflammatory and matrix-remodeling programs; however, direct comparisons of young and aged tumor-associated stromal populations are needed to establish their contribution. Spatial analysis is particularly valuable for determining whether altered fibroblasts and endothelial cells are located near excluded or dysfunctional lymphocytes.

## 5 Immune-stromal circuits that create an aged TME

Immune-stromal circuits are built on bidirectional interactions between immune cells and structural cells such as fibroblasts and endothelial cells. Stromal cells actively exchange signals with immune cells and can sustain local inflammatory or suppressive responses. Among these, fibroblast-myeloid-T-cell interactions are particularly relevant, although their effects vary across tissues and tumors. A recent cross-tissue human fibroblast atlas revealed substantial diversity in fibroblast states and their association with immune modulation (Gao et al., 2024).

In the proposed aged TME niche, senescent fibroblasts may recruit or retain suppressive myeloid cells, whereas myeloid-derived mediators reinforce stromal activation and impair T-cell function. Tregs can further constrain local CD8^+^ T-cell activity. However, a Treg-CD8^+^ T-cell imbalance cannot be inferred from cell numbers alone; suppressive activity, antigen specificity, and spatial proximity are equally critical. These interactions provide a conceptual framework for investigation rather than a universally established sequence of events in aged tumors.

Transforming growth factor-β (TGF-β) exemplifies a stromal pathway relevant to immune exclusion. In metastatic urothelial cancer, a fibroblast TGF-β response signature was linked to resistance against PD-L1 blockade in immune-excluded tumors, and combined pathway inhibition improved T-cell penetration and antitumor activity in a mouse model (Mariathasan et al., 2018). This evidence supports targeting immune-stromal interactions, although it does not prove that TGF-β-mediated exclusion is intrinsically age-dependent. Tumor cell epigenetic programs may also influence antigen presentation and cytokine production, adding further complexity to these circuits.

Environmental factors such as hypoxia, acidity, and altered nutrient availability further shape epigenetic regulation within the TME. Hypoxia can inhibit oxygen-dependent enzymes; for example, reduced TET activity has been associated with tumor DNA hypermethylation (Thienpont et al., 2016). Metabolic pathways determine the availability of substrates and cofactors for chromatin-modifying enzymes, including acetyl-CoA, S-adenosylmethionine, and α-ketoglutarate. Acidity and metabolic stress may further modify these processes, although their effects should not be assumed to mirror those of hypoxia. Currently, it was challenging to experimentally validate interaction especially in human. Whether these environmental changes accelerate aging-specific epigenetic programs rather than simply inducing other stress responses remains an important unresolved question. Therefore, further clinical and translational studies are essential to substantiate these hypotheses.

## 6 Consequences for immunotherapy

Cancer immunotherapy aims to enhance host antitumor immunity and can reshape the TME through both direct and indirect effects (Lv et al., 2022). However, its efficacy varies considerably among patients and cannot be explained by a single parameter. Chronological age has been associated with differences in certain immune checkpoint therapy biomarkers, but these relationships depend on tumor type and biological context (Erbe et al., 2021). Older age should therefore not be treated as a uniform indicator of immunotherapy resistance.

Biological and epigenetic ages are potential complementary biomarkers, yet they are not established as stand-alone predictors of immune checkpoint inhibitor (ICI) response. Systemic aging measures may capture aspects of physiological reserve but fail to reflect local antigen presentation, T-cell state, or stromal exclusion. Conversely, a tumor-derived clock may represent malignant cell biology or cell composition rather than host immune competence. Any proposed biomarker must demonstrate predictive value beyond established clinical and tumor characteristics, ideally in independent cohorts designed to distinguish treatment prediction from general prognosis.

Integrating cell-specific epigenetic measurements with spatial immune states may be more informative than estimating the age of bulk tissue. For example, the coexistence of dysfunctional CD8^+^ T cells, suppressive myeloid cells, and altered stromal regions could identify a local barrier to treatment response. Furthermore, cytokine and growth factor engineering may enhance the efficacy of ICIs and other immunotherapy modalities (Ma and Jamieson, 2025; Wang et al., 2026). However, this possibility remains unvalidated. Similar considerations apply to cancer vaccines, adoptive-cell therapies, and intratumoral immunotherapy. Aging-associated changes may affect antigen presentation, lymphocyte expansion and persistence, and access to tumor cells. Improving one component may be insufficient if other suppressive features of the niche persist.

## 7 Can the aged TME be rejuvenated?

The potential effects of aging-associated TME dysfunction on tumor immunity have prompted interest in strategies to restore immune function. Epigenetic therapies, including DNA methyltransferase (DNMT) inhibitors, histone deacetylase (HDAC) inhibitors, bromodomain and extraterminal (BET) protein inhibitors, and other chromatin-targeting approaches, are under investigation in cancer (Zheng et al., 2025). These agents may alter tumor immunogenicity and immune-cell programs. However, an antitumor effect of an epigenetic drug does not by itself demonstrate reversal of epigenetic aging. Evidence of rejuvenation would require restoration of relevant cellular functions accompanied by appropriately validated molecular changes.

Senolysis, the selective elimination of senescent cells, represents another potential strategy, while senomorphic interventions aim to modify senescence-associated phenotypes without necessarily eliminating the cells (Imawari and Nakanishi, 2024). Their effects depend on the targeted cell population and treatment timing. Senescence can restrain malignant proliferation, yet persistently senescent cells may contribute to an adverse microenvironment. These competing effects argue for selective interventions rather than assuming that all senescent cells should be removed.

Myeloid-derived suppressor cells (MDSCs) and tumor-infiltrating Tregs are also being explored as therapeutic targets (Ibrahim et al., 2025; Tay et al., 2023). CTLA-4-directed antibodies can influence Treg biology, but their clinical antitumor activity should not be uniformly attributed to intratumoral Treg depletion. Human tumor analyses have shown that such depletion is not consistently observed (Sharma et al., 2019). Selectivity is especially important, as systemic inhibition of suppressive immune populations can disrupt tolerance and cause inflammatory toxicity.

The therapeutic goal is therefore to restore effective antitumor immunity while preserving tissue integrity and systemic immune regulation. Local delivery, cell-selective targeting, and carefully timed combinations may help achieve this balance but require direct evaluation in aged models and representative patient cohorts. A reduction in an epigenetic-age estimate alone would be insufficient evidence of clinical benefit. In addition, the lack of methods to fully characterize biological aging in heterogeneous populations makes it difficult to distinguish rejuvenation from the enhancement of tumor immunity, which can normalize the local microenvironment. Therefore, context-dependent effects should be assessed among various cancer cell types and clinical backgrounds.

## 8 Methodological priorities

Factors that modify age-associated phenotypes must be carefully considered when designing clinical studies. Human cancers are highly heterogeneous, and variables such as sex, smoking, alcohol exposure, comorbidities, chronic infections, and prior treatment can influence immune and epigenetic states independently of chronological age. Smoking and alcohol consumption, for example, have been linked to differences in DNA methylation-based aging measures, although these associations are both exposure- and model-dependent (Beach et al., 2015). Accordingly, tumor type, stage, anatomical site, and treatment history should be systematically documented and incorporated into analyses. Paired tumor and non-tumor sampling, combined with longitudinal pre-treatment and post-treatment analyses, can substantially improve interpretation. Adjacent non-tumor tissue provides a useful comparator, although it may already be affected by inflammation, field changes, or prior therapy. Age should be analyzed as a continuous variable whenever possible, with pre-specified cutoffs applied only when clearly justified. Ensuring adequate cohort size and independent validation is essential to minimize the risk of overfitting age-associated signatures.

Integrating single-cell RNA sequencing, single-cell ATAC sequencing, methylation profiling, CITE-seq, and spatial methods can help resolve heterogeneity across TME compartments (Angarola et al., 2025). Each approach measures different features: RNA sequencing identifies transcriptional states, ATAC sequencing assesses chromatin accessibility, and methylation profiling directly examines DNA methylation. These results should not be treated as equivalent evidence for epigenetic aging. When comprehensive single-cell profiling is not feasible, complementary strategies such as cell sorting, computational deconvolution, and tissue-based validation can provide valuable insights. Finally, association-based signatures require functional validation. Experiments should determine whether defined epigenetic alterations affect antigen presentation, immune suppression, lymphocyte cytotoxicity, stromal signaling, or treatment response. Comparisons across young and aged cells or hosts, combined with cell-specific perturbations and rescue experiments, can help disentangle intrinsic aging effects from environmental influences. Careful characterization of human cohorts and experimental models is essential for establishing mechanisms that link epigenetic remodeling to cancer progression and therapeutic efficacy.

## 9 Conclusion

Tumors may harbor epigenetically altered immune-stromal niches whose functional states are not adequately represented by patient age alone. Aging-associated immune dysfunction, stromal senescence, vascular changes, and tumor-induced stress can interact, but they should not be assumed to reflect a single, unified aging program. The next step is to identify which cell-specific epigenetic changes causally sustain these niches and to determine whether modifying them improves treatment response. Future work integrating epigenomics, single-cell profiling, and nutritional or metabolic interventions will be essential to translate these findings into strategies that rejuvenate aged immune niches and enhance therapeutic efficacy. Precision immunotherapy may ultimately benefit from targeting such dysfunctions while preserving protective immune regulation, rather than relying on chronological age alone to guide decisions.
